# Initiation of breastfeeding and prevalence of exclusive breastfeeding at hospital discharge in urban, suburban and rural areas of Zhejiang China

**DOI:** 10.1186/1746-4358-4-1

**Published:** 2009-01-28

**Authors:** Liqian Qiu, Yun Zhao, Colin W Binns, Andy H Lee, Xing Xie

**Affiliations:** 1 Women's Hospital, School of Medicine, Zhejiang University, Hangzhou, Zhejiang, PR China; 2 School of Public Health, Curtin University, Perth, WA, Australia

## Abstract

**Background:**

Rates of exclusive breastfeeding in China are relatively low and below national targets. The aim of this study was to document the factors that influence exclusive breastfeeding initiation in Zhejiang, PR China.

**Methods:**

A cohort study of infant feeding practices was undertaken in Zhejiang Province, an eastern coastal region of China. A total of 1520 mothers who delivered in four hospitals located in city, suburb and rural areas during late 2004 to 2005 were enrolled in the study. Multivariate logistic regression analysis was used to explore factors related to exclusive breastfeeding initiation.

**Results:**

On discharge from hospital, 50.3% of the mothers were exclusively breastfeeding their infants out of 96.9% of the mothers who had earlier initiated breastfeeding. Exclusive breastfeeding was positively related to vaginal birth, baby's first feed being breast milk, mother living in the suburbs or rural areas, younger age of mother, lower maternal education level and family income.

**Conclusion:**

The exclusive breastfeeding rate in Zhejiang is only 50.3% on discharge and does not reach Chinese or international targets. A number of behaviours have been identified in the study that could be potentially incorporated into health promotion activities.

## Background

Breastfeeding has many health and developmental advantages for infants and mothers and is the preferred way of feeding infants to promote optimal infant health and reduced morbidity later in life [1-3]. In Asian cultures, and perhaps more generally, breastfeeding also protects against early *Helicobacter pylori *infection [4-7]. A recent cohort study from Shanghai suggests that breastfeeding may offer a mother some protection against developing Type II diabetes [8]. Breastfeeding has received increased emphasis in China over the past two decades as its importance for child health has become recognized. In order to implement the spirit of the World Summit for Children, the Chinese government issued the "National Programme of Action for Child Development in China in the 1990s" [9]

A number of cross-sectional and longitudinal studies in China showed that the 'ever breastfed' rate, both in urban and rural areas was over 80% in the 1950s and 1960s. During the 1970s and 80s the use of breast milk substitutes became more popular and the national 'ever breastfed' rate decreased, gradually dropping from about 80% in the 1960s to 42.7% in 1975 and it then fell further to 33.6% in 1985 [10]. The trend was even more marked in the large cities such as Shanghai, where the rate fell to 22.2% in 1982 and again to 13% in 1989 [10,11].

The International Baby Friendly Hospital Initiative was introduced with the goal of ensuring that all infants are breastfeeding before their discharge from the hospital and that 80% would be exclusively breastfed for the first six months of life [12]. By 1994, in China a total of 947 hospitals had passed the National Baby Friendly Hospital assessment [13] and since that time the number of BFHI certified hospitals has continued to increase. During the 1990s the Chinese government introduced women and child health protection legislation, society support programs and education programs to support breastfeeding promotion.

Following the introduction of these programs the breastfeeding initiation rate began to rise again. A survey in one of the largest cities of western China, Chengdu, Sichuan province showed that the 'ever breastfed' rate had risen to 88% in 1993 [14]. A longitudinal study found that the 'full breastfeeding rate' was 78% at six weeks in the east coast city of Zibo, Shandong province in 1996 [15]. A survey of mothers from 105 counties showed that by 1995 the breastfeeding initiation rate was well over 90%, but the exclusive breastfeeding rate was low [16]. In a cohort study from the west of China the any breastfeeding rate on discharge was 92% and 73% were continuing to breastfeed at six months [17]. While the trend in breastfeeding rates is encouraging, many of these studies were cross-sectional surveys and have inherent limitations in the information provided on risk factors that could be used in health promotion programs.

Factors that are important in the initiation of breastfeeding include a favourable paternal attitude toward breastfeeding, as perceived by the mother [18], whether the mother had an operative delivery, giving prelacteal feeds and ethnicity [19]. The time that the decision to breastfeed is made, maternal age and education and smoking patterns are also important in some societies [20,21].

Zhejiang province is located in the mid-east coast region of China and has benefited from economic reforms and for the past three decades has had one of the fastest growing regional economies in the country. The economic improvement has created many new job opportunities for the younger generation in high technology industries and has resulted in a large, well educated middle class in the provincial capital of Hangzhou. The rural areas have not benefited as much from the rapid development and people from rural Zhejiang and other provinces continue to move to the capital city and suburban areas in search of a more prosperous city life. In 2006 the provincial population was 49 million with one of the highest population densities in the country. Hangzhou has a population of six million and advertises itself as the "most beautiful city in China" and the many emperors and government officials who have holidayed there in the past bear testament to this fact.

Like other big cities in China, the breastfeeding experience of Zhejiang women has changed over time. A cross-sectional survey undertaken in five cities in Zhejiang in 1997 found that the exclusive breastfeeding rate before discharge was 74.4% and this dropped to 43.7% at 10 weeks [22]. This rate was lower than the World Health Organization target for initiation and for exclusive breastfeeding for six months. In China at the present time the initiation rates of breastfeeding are high and the most important issue is the rate of exclusive breastfeeding [23]. A recent review and study of breastfeeding in rural China confirmed the low rate of exclusive breastfeeding and concluded "health care providers need to intensify education and counseling concerning breastfeeding and especially emphasize the importance of exclusive breastfeeding from birth to 4 to 6 months of age" [24] (p.384). A literature search of the English and Chinese language medical literature failed to find any previous longitudinal studies of breastfeeding in Zhejiang Province prior to this cohort study. The differences in breastfeeding rates in rural, suburban and city areas and the first feeds given in Hangzhou, have previously been described [25,26]. This analysis explores the factors that are important in the initiation of exclusive breastfeeding in Zhejiang Province, PR China.

## Methods

A cohort study of breastfeeding was undertaken in Zhejiang Province, PR China during the period October 2004 to December 2005. The study was designed as a longitudinal study of infant feeding practices with four interviews over a period of six months. In this paper the results from the first two interviews are used in the analysis. The first interview was held one or two days before discharge from hospital. The exact date of discharge and infant feeding information on infant feeding at discharge was obtained from the second interview one month later. The study included mothers from the capital city, Hangzhou, a suburban area on the outskirts of the metropolitan area, Fu-yang, and a mountainous rural area approximately 300 km to the southwest. A total of 1520 mothers were recruited from hospitals in each location (two in the rural area). Each of these hospitals is typical of the health care facilities in the area they are located. All the mothers in the study were recruited while in hospital and in the larger facilities where there were a number of deliveries on one day, a system of random numbers was used to select the mothers to be included.

The project was approved by the Zhejiang local research authorities (Zhejiang University, Women's Hospital Ethics Committee) and the Human Research Ethics Committee of Curtin University, Australia. Mothers who agreed to participate in the study signed the consent page attached to the questionnaire and were informed of their rights to withdraw from the follow-up process at anytime without prejudice. They were assured that all of the personal data collected would be kept confidential and identifying data removed from the computer files.

The first interview was undertaken in person by a nurse or women's health worker before discharge from hospital and follow-up interviews were held at one month, three months and six months post partum. Most of the follow-up interviews (92%) were undertaken by telephone, a testimony to the rapid economic development of this province. In the few instances where mothers could not be reached by telephone, the follow-up interviews were completed at the routine examinations in the community child care clinics.

The study sample consisted of 1520 mothers who were recruited from the three locations. The inclusion criteria were that the mother had delivered a live child, the mother and neonate did not have serious diseases and that she was resident in the service area of that hospital. Mothers who were not local residents were not included in the study. While in Hangzhou this included almost one half of the deliveries, in the suburban and rural areas almost all mothers were local residents and were eligible to participate. The response rate was high and 98% of mothers (1520 out of 1551) agreed to participate in the study.

The questionnaire included full details of infant feeding methods and factors likely to influence the initiation and duration of breastfeeding and was based on questionnaires that have been extensively used in breastfeeding cohort studies in China, Australia, Vietnam and Kenya [19,27-30]. The questionnaires were translated and were then tested in focus groups to ensure cultural appropriateness.

All data analyses were carried out using the Statistical Package for Social Sciences (SPSS), release 14.0 (SPSS Inc., Chicago, IL, USA). Multivariate logistic regression was used to determine factors associated with exclusive breastfeeding at discharge. Backward elimination procedure was applied to remove those variables with non-significant effect.

Definitions used in this study were from Xu and colleagues [17] and the WHO definition of exclusive breastfeeding [31]:

### 'Any breastfeeding'

The child has received breastmilk (direct from the breast or expressed) with or without other drinks, formula or other infant food.

### 'Exclusive breastfeeding'

Breastfeeding while giving no other food or liquid, not even water, within 24 hours of interview, with the exception of drops or syrups consisting of vitamins, mineral supplements or medicine.

## Results

The details of the sample are shown in Table 1 with the relationship between demographic factors and exclusive breastfeeding rates. The 'any breastfeeding' rate at discharge from hospital was 96.9%. No significant differences in the 'any breastfeeding' rates were found between the city, suburban and rural areas. The exclusive breastfeeding rate on discharge in Zhejiang was 50.3%, but differed significantly between the three locations: 38% in the city and 63.4% and 61% in the suburban and rural areas [26]. The average hospital stay was 6.4 days and 93% of women were discharged before eight days. The average stay in hospital following a caesarean section was 6.9 days, compared to 5.3 days following vaginal delivery. In the univariate analysis in Table 1 the other significant factors were maternal age and education, having a caesarean section, living with parents, the time the decision to breastfeed was made, parity, attendance at antenatal class, monthly income and whether breast milk was the first feed given to the infant.

**Table 1:** Initiation of breastfeeding and prevalence of exclusive breastfeeding at hospital discharge

			**Exclusive breastfeeding**			
					
	**Total N of women**	**%**	**n**	**%**	**Unadjusted Odds Ratio**	**95% CI**
**Resident**						
City	635	41.9	233	38.0	1	
Suburb	347	22.9	213	63.4	2.82	2.14, 3.72
Rural	532	35.2	316	61.0	2.55	2.01, 3.24
**Maternal age**						
<25	358	23.9	225	64.8	1	
25–29	800	53.5	379	48.6	0.51	0.39, 0.67
≥ 30	338	22.6	148	45.8	0.46	0.34, 0.63
**Caesarean section**						
No	495	32.7	282	58.6	1	
Yes	1019	67.3	480	48.7	0.67	0.54, 0.83
**Maternal education (years)**						
≤ 9 years	544	36.0	330	62.7	1	
10–12 years	370	24.5	178	49.4	0.58	0.44, 0.76
> 12 years	597	39.5	251	43.4	0.46	0.36, 0.58
**Baby's gender**						
Male	772	51.3	378	50.5	1	
Female	732	48.6	379	53.5	1.13	0.92, 1.39
**Time breastfeeding decision made**						
Before pregnancy	1089	72.3	564	53.2	1	
During pregnancy	263	17.5	136	54.2	1.04	0.79, 1.37
After baby born	154	10.2	59	39.9	0.58	0.41, 0.83
**Parity**						
Primiparous	1347	89.2	655	50.2	1	
Multiparous	163	10.8	104	65.4	1.87	1.33, 2.64
**Gestational age (weeks)**						
<37	49	3.3	18	41.9	1	
≥ 37	1443	96.7	731	52.1	1.51	0.82, 2.80
**Living with parents**						
Yes	742	49.4	414	57.4	1	
No	759	50.6	346	47.1	0.66	0.54, 0.81
**First feed**						
Breast milk	928	62.5	499	53.9	1	
Other	557	37.5	245	47.7	0.78	0.63, 0.97
**Mother's employment**						
Laborer	492	33.3	295	61.3	1	
Office work	763	51.7	330	44.7	0.51	0.40, 0.64
Not employed	221	15.0	110	52.4	0.69	0.50, 0.96
**Attended antenatal class**						
Yes	949	63.1	445	48.0	1	
No	555	36.9	310	58.5	1.53	1.23, 1.89
**Birth weight**						
<2500	27	1.8	14	58.3	1	
2500–3999	1382	91.8	700	52.1	0.78	0.34, 1.76
≥ 4000	97	6.4	42	45.7	0.60	0.24, 1.49
**Admitted to NICU**						
Yes	147	9.9	55	40.4	1	
No	1333	90.1	687	52.9	1.66	1.16, 2.37
Family monthly income (RMB)*						
≤ 1500	215	14.6	135	64.6	1	
1501–3000	401	27.2	236	59.9	0.82	0.58, 1.16
3001–5000	451	30.6	195	45.1	0.45	0.32, 0.63
>5000	407	27.6	170	43.1	0.42	0.29, 0.59
**Grandmother breastfed**						
Yes	1408	96.0	705	51.7	1	
No	58	4.0	39	51.3	0.98	0.62, 1.56

The exchange rate at the time of the study was RMB 7.5. = 1 USD, RMB 10.6 = 1 Euro.

Differences in the mother's feeding practices and the factors which may influence feeding practices before discharge for city, suburban and rural areas are detailed in Table 2. In this study many mothers, overall 41.4%, believed that their breast milk was insufficient to feed their babies. This perception was worse in the city, where 47.9% of mothers felt they lacked enough breast milk compared to 34.4% in the suburb and 38% in the rural areas respectively.

**Table 2:** The mother's feeding practices in city, suburb and rural areas, Zhejiang (2004–2005)

		**City**		**Suburb**		**Rural**		**Total**	
**Variable**		**n**	**%**	**N**	**%**	**n**	**%**	**n**	**%**
First feed	Breast milk	472	74.3	247	71.6	209	41.4	928	62.5
	Not breast milk	163	25.7	98	28.4	296	58.6	557	37.5
	Missing	3		2		30		35	

Time breastfeeding decision made	Before pregnancy	482	75.9	255	73.5	352	67.2	1089	72.3
	During pregnancy	103	16.2	73	21.0	87	16.6	263	17.5
	After baby born	50	7.9	19	5.5	85	16.2	154	10.2
	Unknown	3		0		11		14	

Mother received gift of formula	Yes	246	38.7	88	25.4	157	31.8	491	33.3
	No	389	61.3	258	74.6	337	68.2	984	66.7
	Unknown	3		1		41		45	

First breastfeed	≤ 30 min	163	27.0	178	53.5	142	27.8	483	33.4
	>30 min	441	73.0	155	46.5	369	72.2	965	66.6
	Unknown	34		14		24		72	

Colostrum secretion	≤ 1 day	403	64.1	164	47.4	182	36.2	749	50.7
	≥ 2 days	226	35.9	182	52.6	321	63.8	729	49.3
	Unknown	9		1		32		42	

Baby admitted to NICU	Yes	106	16.7	19	5.5	22	4.4	147	9.9
	No	528	83.3	326	94.5	479	95.6	1333	90.1
	Unknown	4		2		34		40	

The factors that could be involved in exclusive breastfeeding were incorporated into a multivariate logistic regression model. When adjusted for potential confounding factors, the factors which were related to exclusive breastfeeding are described in Table 3. The factors that significantly contributed to decreasing the likelihood of exclusive breastfeeding included mothers who had a caesarean section (OR = 0.76). Demographic factors that were positively associated with exclusive breastfeeding at discharge were living in the suburb (OR = 2.17) and rural areas (OR = 2.33). Mothers who were older than 24 years (OR = 0.58, OR = 0.51), who did not make the decision to breastfeed until after birth (OR = 0.57) and who didn't give breastmilk as the first feed (OR = 0.56) were less likely to be exclusively breastfeeding on discharge.

**Table 3:** Factors associated with exclusive breastfeeding initiation after adjustment for potential confounders in Zhejiang Province, China, 2004–2005

**Factors**		**n**	Adjusted Odds Ratio*	**95%CI**	
Delivery method	Vaginal	407	1		
	Caesarean	865	0.76	0.59	0.99
First feed	Breast milk	828	1		
	Other	444	0.56	0.43	0.73
Living place	City	599	1		
	Suburb	318	2.17	1.59	2.95
	Rural	355	2.33	1.69	3.21
Maternal age	<25	298	1		
	25–29	684	0.58	0.43	0.79
	≥ 30	290	0.51	0.34	0.75
Breastfeeding decision	Before pregnancy	946	1		
	During pregnancy	201	1.03	0.75	1.43
	After birth	125	0.57	0.38	0.86
Parity	Primiparous	1142	1		
	Multiparous	130	1.67	1.08	2.57

∘ All variables of interest were included in the full model in the initial step and then backward elimination procedure was applied to obtain the final model, using 5% critical value of χ^2 ^test for the appropriate degrees of freedom. * -2 log likelihood = 1637.86, d.f = 11

∘ Non-significant variables were maternal age, cesarean section, maternal education, infants' gender, when decided feeding method, parity, first feed, gestation week, mothers' job, if mothers attended antenatal classes, infant birth weight, if infants admitted to special care nursery? Living place, family income, maternal grandmother breastfed? Living with other people or only the couple.

## Discussion

The use of prelacteal feeds were common in all locations (37.5%) and their use in Hangzhou city have been described in more detail [25]. Their use was most common in the rural location where it is traditional not to breastfeed for some time after birth. This is also the case in the far west of China, where delayed first feeds were common, but the use of prelacteal feeds was not as high as in Hangzhou [23]. In Vietnam the use of fluids other than breastmilk as a first feed is also common, but there it is less likely to be infant formula [29]. All of the hospitals in our study are "Baby Friendly Hospital" accredited, as are most Chinese hospitals. In theory all hospitals are required to practice the WHO Ten Steps to Successful Breastfeeding. Practically, the hospitals find that there are some difficulties in following these steps strictly. If a mother (or often her family) feels she does not have enough milk, they can easily get infant formula either from her family or from the hospital.

In this study there are several factors that could be potentially modified to increase exclusive breastfeeding rates. The length of time to the first feed is an important factor and is used to monitor progress towards the millennium goals for child health [32]. In this study only one third (33.4%) of infants began breastfeeding within 30 minutes of delivery. Delivery room practices need to be modified to make this possible.

According to Chinese tradition, it is the practice of Chinese friends or relations of postpartum women to visit the mother and they bring gifts which could be consumed or worn by the new baby. In recent times infant formula has become the most popular gift for new mothers. Gifts of infant formula were given to one third of new mothers by friends or relations (see Table 2).

The time that the decision is made to breastfeed has an important relationship to breastfeeding outcomes in a number of different cultures [18,33]. This is a function of antenatal preparation and health professionals need to encourage prospective parents to think about the importance of breastfeeding at the earliest opportunity and to continue to discuss this at subsequent contacts. Assisting mothers to make an early decision could also include education of the infant's father and grandmother about the benefits of breastfeeding.

Exclusive breastfeeding rates were lower in infants who were delivered by caesarean section. This is a common risk factor for not breastfeeding in Asian societies, but not in Australia [29,34,35]. In our study population, women having a caesarean section had lower rates of exclusive breastfeeding than with vaginal delivery. After surgery, mothers feel pain in their abdominal incision, movement is limited because of catheterization and intravenous lines, and Chinese mothers worry about the side effects of medicines which may pass to the baby via breast milk. These factors are believed in this culture to influence lactogenesis. Further studies are needed to obtain more details on the reasons for the higher prevalence of caesarean section in this population and the influence on breastfeeding.

There are several limitations that need to be considered when interpreting the results of this study. The sample was restricted to three locations in Zhejiang Province. While these locations were selected to be representative of Zhejiang and the response rate was high, this should be born in mind when interpreting the results. As economic and health system developments occur in Zhejiang Province it will be important to repeat cohort studies so that breastfeeding is continued to be promoted.

## Conclusion

In Zhejiang Province the exclusive breastfeeding rate on discharge from hospital was only 50.3%, ranging from a low of 38% in the city to 63% in the suburbs. Risk factors for not exclusively breastfeeding include having a caesarean section, the time at which the decision to breastfeed was made, the place of residence and whether a prelacteal feed was given. Biological factors included maternal age and parity. Some of these factors could be incorporated into trials to increase exclusive breastfeeding rates.

## Competing interests

The authors declare that they have no competing interests.

## Authors' contributions

All authors contributed to the study. LQ designed the research, collected and analyzed data, drafted the manuscript. YZ analyzed data and revised the manuscript. CWB designed the research, drafted and revised the manuscript. AHL analyzed data and revised the manuscript. XX designed the research, collected data and revised the manuscript.

## References

[B1] Jones G, Steketee R, Black R, Bhutta Z, Morris S, the Bellagio Child Survival Study Group (2003). How many child deaths can we prevent this year?. Lancet.

[B2] Binns C, Davidson G (2003). Infant Feeding Guidelines for Health Workers. Dietary Guidelines for Children in Australia.

[B3] WHO Collaborative Study Team on the Role of Breastfeeding on the Prevention of Infant Mortality (2000). Effect of breastfeeding on infant and child mortality due to infectious diseases in less developed countries: a pooled analysis. Lancet.

[B4] Horta BL, Bahl R, Martines JC, Victora CG (2007). Evidence on the long-term effects of breastfeeding: systematic review and meta-analyses.

[B5] Okuda M, Miyashiro E, Koike M, Okuda S, Minami K, Yoshikawa N (2001). Breast-feeding prevents Helicobacter pylori infection in early childhood. Pediatrics International.

[B6] Okuda M, Miyashiro E, Booka M, Tsuji T, Nakazawa T (2007). Helicobacter pylori colonization in the first 3 years of life in Japanese children. Helicobacter.

[B7] Pearce MS, Thomas JE, Campbell DI, Parker L (2005). Does increased duration of exclusive breastfeeding protect against Helicobacter pylori infection? The Newcastle thousand families cohort study at age 49–51 years. J Pediatr Gastroenterol Nutr.

[B8] Villegas R, Gao YT, Yang G, Li HL, Elasy T, Zheng W, Shu XO (2008). Duration of breast-feeding and the incidence of type 2 diabetes mellitus in the Shanghai Women's Health Study. Diabetologia.

[B9] He J, Wang F (1994). Baby friendly action in China.

[B10] Wang F, Zhu Z, Tong F (1991). Discussion and suggestion about promotion national breastfeeding. Maternal and Child Health Care of China.

[B11] Chen J, Ji P (1993). Process to promote breastfeeding in Beijing. Maternal and Child Health Care of China.

[B12] Naylor AJ (2001). Baby-Friendly Hospital Initiative. Protecting, promoting, and supporting breastfeeding in the twenty-first century. Pediatr Clin North Am.

[B13] Huang X (1995). The summary of the national breastfeeding symposium. Chinese Journal of Obstetrics and Gynecology.

[B14] Guldan GS, Zhang M, Zeng G, Hong J, Yang Y (1995). Breastfeeding practices in Chengdu, Sichuan, China. J Hum Lact.

[B15] Zhang LY, Liu YR, Shah IH, Tian KW, Zhang LH (2002). Breastfeeding, amenorrhea and contraceptive practice among postpartum women in Zibo, China. Eur J Contracept Reprod Health Care.

[B16] Wang X, Wang Y, Kang C (2005). Feeding practices in 105 counties of rural China. Child: Care, Health and Development.

[B17] Xu F, Binns C, Wu J, Yihan R, Zhao Y, Lee A (2007). Infant feeding practices in Xinjiang Uygur Autonomous Region, People's Republic of China. Public Health Nutr.

[B18] Scott JA, Binns CW, Graham KI, Oddy WH (2006). Temporal changes in the determinants of breastfeeding initiation. Birth.

[B19] Xu F, Binns C, Yu P, Bai Y (2007). Determinants of breastfeeding initiation in Xinjiang, PR China, 2003–2004. Acta Paediatr.

[B20] Gottschang SZ (2007). Maternal bodies, breast-feeding, and consumer desire in urban China. Med Anthropol Q.

[B21] Scott JA, Binns CW (1999). Factors associated with the initiation and duration of breastfeeding: a review of the literature. Breastfeed Rev.

[B22] Qiu L, Wang Q, Li R (1998). The survey of breastfeeding in city, Zhejiang Province. Zhejiang Prevention Medicine.

[B23] Xu F, Binns C, Zheng S, Wang Y, Zhao Y, Lee A (2007). Determinants of exclusive breastfeeding duration in Xinjiang, PR China. Asia Pac J Clin Nutr.

[B24] Shi L, Zhang J, Wang Y, Guyer B (2008). Breastfeeding in rural china: Association between knowledge, attitudes and practices. J Hum Lact.

[B25] Qiu L, Xie X, Lee A, Binns CW (2007). Infants' first feeds in Hangzhou, PR China. Asia Pac J Clin Nutr.

[B26] Qiu L, Zhao Y, Binns CW, Lee AH, Xie X (2008). A cohort study of infant feeding practices in city, suburban and rural areas in Zhejiang Province, PR China. Int Breastfeed J.

[B27] Scott JA, Landers MC, Hughes RM, Binns CW (2001). Factors associated with breastfeeding at discharge and duration of breastfeeding. J Paediatr Child Health.

[B28] Scott JA, Aitkin I, Binns CW, Aroni RA (1999). Factors associated with the duration of breastfeeding amongst women in Perth, Australia. Acta Paediatr.

[B29] Duong DV, Binns CW, Lee AH (2004). Breast-feeding initiation and exclusive breast-feeding in rural Vietnam. Public Health Nutr.

[B30] Lakati A, Binns C, Stevenson M (2002). The effect of work status on exclusive breastfeeding in Nairobi. Asia Pac J Public Health.

[B31] WHO Division of Diarrhoeal and Acute Respiratory Disease Control (1991). Indicators for assessing breastfeeding practices.

[B32] WHO (2003). Infant and Young Child Feeding: A tool for assessing national practices, policies and programmes.

[B33] Duong DV, Lee AH, Binns CW (2005). Determinants of breast-feeding within the first 6 months post-partum in rural Vietnam. J Paediatr Child Health.

[B34] Chen LH, Liu CK, Merrett C, Chuo YH, Wan KS (2008). Initiation of breastfeeding lessons from Taiwan. Paediatric Nursing.

[B35] Chung W, Kim H, Nam CM (2008). Breast-feeding in South Korea: factors influencing its initiation and duration. Public Health Nutr.

